# Drug Repurposing Based on Protozoan Proteome: In Vitro Evaluation of In Silico Screened Compounds against *Toxoplasma gondii*

**DOI:** 10.3390/pharmaceutics14081634

**Published:** 2022-08-05

**Authors:** Débora Chaves Cajazeiro, Paula Pereira Marques Toledo, Natália Ferreira de Sousa, Marcus Tullius Scotti, Juliana Quero Reimão

**Affiliations:** 1 Laboratory of Preclinical Assays and Research of Alternative Sources of Innovative Therapy for Toxoplasmosis and Other Sicknesses (PARASITTOS), Departamento de Morfologia e Patologia Básica, Faculdade de Medicina de Jundiaí, Jundiaí 13202-550, Brazil; 2 Programa de Pós-graduação em Produtos Naturais e Sintéticos Bioativos (PgPNSB), Instituto de Pesquisa em Fármacos e Medicamentos (IPeFarM), Universidade Federal da Paraíba, Campus I, Cidade Universitária, João Pessoa 58051-900, Brazil

**Keywords:** bioinformatics, drug repurposing, toxoplasmosis, *Toxoplasma gondii*, in vitro screening, drug targets, drug discovery

## Abstract

*Toxoplasma gondii* is a protozoan that infects up to a third of the world’s population. This parasite can cause serious problems, especially if a woman is infected during pregnancy, when toxoplasmosis can cause miscarriage, or serious complications to the baby, or in an immunocompromised person, when the infection can possibly affect the patient’s eyes or brain. To identify potential drug candidates that could counter toxoplasmosis, we selected 13 compounds which were pre-screened in silico based on the proteome of *T. gondii* to be evaluated in vitro against the parasite in a cell-based assay. Among the selected compounds, three demonstrated in vitro anti-*T. gondii* activity in the nanomolar range (almitrine, bortezomib, and fludarabine), and ten compounds demonstrated anti-*T. gondii* activity in the micromolar range (digitoxin, digoxin, doxorubicin, fusidic acid, levofloxacin, lomefloxacin, mycophenolic acid, ribavirin, trimethoprim, and valproic acid). Almitrine demonstrated a Selectivity Index (provided by the ratio between the Half Cytotoxic Concentration against human foreskin fibroblasts and the Half Effective Concentration against *T. gondii* tachyzoites) that was higher than 47, whilst being considered a lead compound against *T. gondii*. Almitrine showed interactions with the Na^+^/K^+^ ATPase transporter for *Homo sapiens* and *Mus musculus*, indicating a possible mechanism of action of this compound.

## 1. Introduction

*Toxoplasma gondii* is an obligate intracellular protozoan parasite that belongs to Apicomplexa Phylum and is the etiological agent of toxoplasmosis [1]. The parasite diverged from closer species due to its ability to infect a wide range of hosts, re-enforced by flexible transmission pathways [2]. Because of this, it is estimated that more than 60% of the population throughout the world have been infected [3] and, in Brazil, the serologic prevalence of *T. gondii* human infection ranges from 50% to 80% [4].

Despite the importance of toxoplasmosis to public health, considering its high prevalence in the human population and the serious clinical manifestations, mainly in immunocompromised patients and in cases of congenital infection [5], there are still very few therapeutic options available, these being effective only against the acute form of the disease [6].

Ideal drugs for toxoplasmosis treatments should be effective against the chronic form of infection and be offered at an affordable price, and present low or zero toxicity [7]. Ideally, they should also not present risks of congenital malformation, allowing pregnant women to use them freely. However, several of these characteristics are not found in drugs currently used in standard toxoplasmosis therapy, which has remained unchanged since the beginning of the 1990s [7]. Since current chemotherapy is insufficiently effective, with extended treatments that vary from weeks to over a year in duration, or show high toxicity [8], alternative therapeutic options for toxoplasmosis treatment are of utmost importance.

The research and development of new drugs represent a slow and onerous process. New techniques have been proposed to speed up this process, and one of these is called ‘drug repositioning’. It consists of a strategy that seeks new applications for an existing drug, which have not been previously referenced and are not currently prescribed or researched [9].

Aiming to find new uses for already known compounds, the international organization Medicines for Malaria Venture (MMV) and the Drugs for Neglected Diseases initiative (DNDi), together with researchers from the industrial and academic fields, created the Pandemic Response Box and the COVID Box. Together, these collections consist of 560 structurally diverse active compounds, all set for trial against infectious and neglected diseases. These compounds were selected from an extensive list of antibacterial, antifungal, and antiviral compounds, all of which are already being commercialized or are in the clinical development phase [10].

Malaria Box and Pathogen Box are two other collections created by MMV that gather around 800 compounds with confirmed activity against the most socio-economic relevant diseases all over the world, such as malaria, tuberculosis, sleeping sickness, leishmaniasis, schistosomiases, ancylostomiasis, toxoplasmosis, cryptosporidiosis, and dengue. These collections were used to identify new drug candidates for the treatment of many diseases, including toxoplasmosis [2,11,12].

Databases of bioactivity, such as ChEMBL and DrugBank, provide information about the interaction between compounds and proteins. Sarteriale et al. [13] have presented an approach to pre-track the entire proteome of any organism with available genomic data against known drug targets, using a combination of Ruby scripts and freely available resources. This method was used to predict inhibitors for disease-causing protozoan parasites. The authors performed the in vitro validation of the in silico results obtained, using a cell-based *Cryptosporidium parvum* growth assay, showing that the predicted inhibitors were significantly more likely than those expected randomly by chance. However, the identified compounds had not yet been evaluated against *T. gondii* in a cell-based assay until now. Here, we tested some of the inhibitors identified by Sarteriale et al. 2014 [13], aiming to confirm the in silico predicted activity against *T. gondii* in a cell-based assay. 

**Figure 1:**
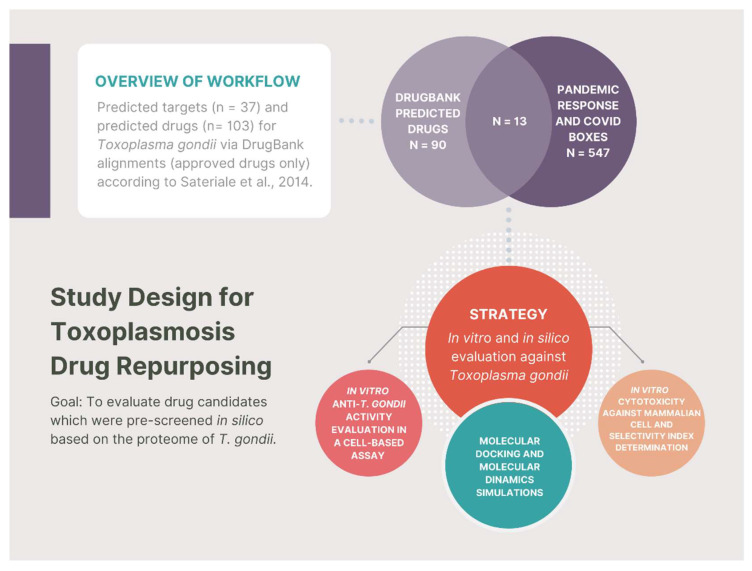
Study design and workflow. Following the publication of predicted drugs for *T. gondii* via DrugBank alignments by Sarteriale et al., (2014) [13], we selected 13 compounds from Pandemic Response and COVID Boxes to be in vitro evaluated against *T. gondii* and for Molecular Docking and Dinamics Simulations in the present work. Among the 13 selected compounds, three demonstrated in vitro anti-*T. gondii* activity in the nanomolar range (almitrine, bortezomib, and fludarabine), and ten compounds demonstrated anti-*T. gondii* activity in the micromolar range.

Amongst the compounds that presented a *T. gondii* protein as their target in the virtual screening, 13 were selected for evaluation against *T. gondii* in the present work. This selection was based on the presence of these compounds in the MMV Pandemic Response Box and COVID Box collections, aiming to evaluate in vitro the predicted activity against *T. gondii* (Figure 1).

We crossed the in silico screening results achieved by Sarteriale et al. 2014 [13] with the MMV libraries, aiming to build a small enriched compound collection for in vitro drug testing.

Our objective was to test only the in silico predicted *T. gondii* inhibitors available in the Pandemic Response Box and COVID Box collections, enabling a more efficient use of laboratory resources. We obtained 100% accuracy, since all these 13 compounds showed anti-*T. gondii* activity in the micromolar or nanomolar range, this being the first report about the in vitro anti-*T. gondii* activity of almitrine, bortezomib, and fludarabine.

Drug development is a lengthy, complex, and costly process, entrenched with a high degree of uncertainty that a drug will succeed. In this context, drug repurposing—A strategy for identifying new clinical uses for existing drugs—Becomes an interesting strategy for drug discovery, as it involves potentially lower financial costs in drug development as well as shorter timelines [14].

Because repurposing screens can be costly and time consuming, an in silico drug screen with the ability to identify drugs with a high likelihood of activity improves the chances of success by enabling the pre-selection of compounds to test in vitro. 

Here we connected traditional drug discovery techniques with computer-based tools to deliver robust drug repurposing hints. We used a target-based pre-screen that utilized simple sequence alignment techniques to discover potential drugs [13]. Drugs’ structural and physicochemical properties and the predicted drug-target interactions were explored to select potential re-positioned compounds to treat toxoplasmosis. Therefore, the contributions of this manuscript are:

- To demonstrate the in vitro anti-*T. gondii* activity of the 13 pre-screened compounds: almitrine, bortezomib, digitoxin, digoxin, doxorubicin, fludarabine, fusidic acid, levofloxacin, lomefloxacin, mycophenolic acid, ribavirin, trimethoprim, and valproic acid;
- To demonstrate the in vitro cytotoxic of almitrine, bortezomib and fludarabine against human foreskin fibroblasts;
- To investigate the mechanism of action of the 13 compounds using Molecular Docking through the binding affinity of the compound and the predicted molecular target;
- To carry out Molecular Dynamics simulations of almitrine to assess the flexibility of the transporting ATPase alpha 1 and the stability of the enzyme interactions in the presence of factors such as solvent, ions, pressure and temperature.

The goal of the present work is therefore to contribute to the discovery of new candidates for toxoplasmosis chemotherapy, using repositioned compounds. The strategy of drug repositioning allows for efficient progress in the drug discovery process since many of the compounds are clinically safe and have well established pharmacological action.

## 2. Materials and Methods

### 2.1. Drugs and Chemicals

Pyrimethamine (PYR), dimethyl sulfoxide (DMSO), chlorophenol red-β-D-galactopyranosidase (CPRG), phosphate buffer saline (PBS) and 3-[4,5-dimethylthiazol-2-yl]-2,5-diphenyltetrazolium bromide (MTT) were purchased from Sigma-Aldrich Corporation. Dulbecco’s Modified Eagle’s Medium (DMEM), fetal bovine serum (FBS), dithiothreitol (DTT), HEPES and sodium dodecyl sulfate (SDS) were purchased from Thermo Fisher Scientific. Pandemic Response Box (PRB) and COVID Box (CB) were kindly donated by the Medicines for Malaria Venture (MMV) foundation. Other analytical reagents were purchased from Sigma-Aldrich, unless otherwise stated.

### 2.2. Cell Culture and Parasite Propagation

Tachyzoites of the RH strain encoding a transgenic copy of β-galactosidase (type I, clone 2F1) [15] were continually passaged in confluent monolayers of human foreskin fibroblasts (HFF), cultured in DMEM supplemented with 2% FBS (D2 medium), L-glutamine (2 mM) and gentamycin (10 μg/mL) [16]. Fresh emerging tachyzoites were counted, diluted in a fresh culture medium, and added to 96-well plates containing HFF monolayers as described below. All HFF and parasite cultures were grown in a 37 °C incubator supplemented with 5% CO_2_.

### 2.3. β-Galactosidase-Based Growth Inhibition Assays

Firstly, 5 × 10^3^ HFF cells/well (in 100 μL volume) were placed in 96-well plates and incubated overnight to adhere. Afterwards, the wells were emptied and refilled with fresh D2 medium containing 5 × 10^3^ RH-2F1 parasites (in 100 μL volume) and incubated for 3 h at 37 °C, 5% CO_2_. Subsequently, compounds were serially diluted in D2 medium and added to the infected plates and incubated for 72 h at 37 °C, 5% CO_2_. Each drug concentration was assessed in two replicate wells. Finally, β-galactosidase activity was evaluated as previously described [17]. Infected cells were incubated with 100 µL of lysis buffer (100 mM HEPES, 1 mM MgSO_4_, 0.1% Triton X-100, 5 mM DTT) for 15 min. Afterwards, the lysates were mixed with 160 µL of assay buffer (100 mM phospate buffer pH 7.3, 102 mM β-mercaptoethanol, 9 mM MgCl_2_) and, subsequently, with 40 µL of 6.25 mM CPRG. After incubating the reaction mixtures for 30 min, the β-galactosidase activity was measured at 570 nm using a microplate reader (Thermo Scientific™ Varioskan LUX). Pyrimethamine was used as a reference drug (positive control) in all assays. Data presented are representative of the results of two or more biological replicates. Dose-response inhibition curves (Log (inhibitor) vs. normalized response—Variable slope) were obtained using Skanlt Software (Thermo Scientific, Waltham, MA, USA).

### 2.4. Cytotoxicity in Mammalian Cells

HFF were seeded at 5 × 10^4^ cells/well in 96-well microplates and incubated overnight to adhere to the plate. After that, the cells were incubated in the presence of increasing concentrations of the compounds for 72 h at 37 °C in a 5% CO_2_ humidified incubator. The viability of the cells was determined by the MTT assay as previously described [18]. The medium in each well was replaced by PBS (100 µL/well), MTT (5 mg/mL) was added (20 µL/well), and the plate was incubated for 4 h at 37 °C. Formazan extraction was performed using 10% SDS for 18 h (80 µL/well) at room temperature, and the optical density was measured at 550 nm using a microplate reader (Thermo Scientific™ Varioskan LUX). HFF incubated in D2 without drug treatment were used as viability control. Viability of 100% was expressed based on the optical density of untreated HFF cells, after normalization. The Selectivity Index (SI) was provided by the ratio between the CC_50_ against HFF cells and the EC_50_ against *T. gondii* tachyzoites. Data presented are representative of the results of two or more biological replicates. Dose-response inhibition curves (Log (inhibitor) vs. normalized response—Variable slope) were obtained using Skanlt Software (Thermo Scientific).

### 2.5. Molecular Docking

Molecular Docking was used to investigate the mechanism of action of the 13 compounds included in the study that contribute to the inhibitory effect of *T. gondii* through the binding affinity of the compound and the predicted molecular target [19]. The 3D structure of the enzyme was obtained from the Protein Data Bank (PDB) (https://www.rcsb.org/pdb/home/home.do accessed on 14 March 2022) [20]. Initially, all water molecules were removed from the crystalline structure and the root mean square deviation (RMSD) was calculated from the postures, indicating the degree of reliability of the fit. RMSD provides the connection mode close to the experimental structure and is considered successful if the value is less than 2.0 Å [21]. We used two softwares—Molegro Virtual Docker v.6.0.1 (MVD) (CLC Bio Company, Aarhus, Denmark) and PYRX—Virtual Screening Tool, Source Force, 2022, Slashdot Media. The complexed ligand was used to define the active site. The compound was then imported to analyze the stability of the system through the interactions identified with the active site of the enzyme, taking as a reference the energy value of the MolDock Score [22]. The MolDock SE (Simplex Evolution) algorithm was used with the following parameters: A total of 10 runs with a maximum of 1500 iterations, using a population of 50 individuals, with 2000 minimization steps for each flexible residual and 2000 global minimization steps per run. The MolDock score (GRID) scoring function was used to calculate docking energy values. A GRID was set at 0.3 A and the search sphere was set at 15 A in radius. For the analysis of ligand energy, internal electrostatic interactions, internal hydrogen bonds and sp2-sp2 torsions were evaluated [23,24]. The PYRX—Virtual Screening Tool, Source Force, 2022, Slashdot Media features two main programs, corresponding to: Auto Dock (version 4.2.6), (Center for Computational Structural Biology, San Diego, CA, USA) which uses force fields such as AMBER in conjunction with free energy scoring functions, plus affinity maps and pre-calculated electrostatic maps for specific atoms [25,26]. The second program refers to Auto Dock Vina (version 1.2), (Center for Computational Structural Biology, San Diego, CA, USA), which corresponds to a more recent and improved version of the calculation platform. The software uses a semi-flexible docking algorithm by default. The anchoring site of the receptor being defined within the binding site of the co-crystallized ligand, identified through the coordinates of the ligand after importing and labeling the macromolecule [27,28]. The program was used with a default plug-in parameter. Furthermore, the hydrogen bonding distance (O-H) was defined at <2.50 Ǻ between the donor and acceptor atoms with a minimum hydrogen donor-acceptor angle of 120°. Grid size was adjusted to 25 Ǻ in each dimension. The proteins used in the study were, respectively: thymidyl synthase in complex with 2-amino-5-(phenylsulfanyl)-3,9-dihydro-4H-pyrimido[4,5-b]indol-4-one (PDB: 4KY4) [29], purine nucleoside phosphorylase in complex with 1,4-dideoxy-4-aza-1-(s)-(9-deazahypoxanthine-9-yl)-d-ribitol (PDB: 3MB8) [30], enoyl-acyl carrier protein reductase (ENR) in complex with triclosan (PDB: 2O2S) [31], and calcium dependent protein kinase 1 in complex with 5-amino-1-tert-butyl-3-(quinolin-2-yl)-1H-pyrazole-4-carboxamide (PDB: 4M84) [32]. In addition, to evaluate the specificity of the mechanism of action with Na^+^/K^+^-transporting ATPase alpha 1, the construction of this macromolecule was carried out for the species *Homo sapiens* and *Mus musculus* [32] with thapsigargin [33] as a positive control.

#### Docking Consensus

To increase the accuracy of the results obtained, a Docking consensus analysis was performed in order to provide a better selection of the compounds under study. Regarding the Molegro Virtual Docker v.6.0.1 (MVD) program (CLC Bio Company, Aarhus, Denmark), the values of the Moldock Score and PlantScore algorithms were used. Regarding the PYRX program—Virtual Screening Tool, Source Force, 2022, Slashdot Media, AutoDock Vina (version 1.2) (Center for Computational Structural Biology, San Diego, CA, USA) was used.

The determination of the affinity of the 13 compounds under study for the targets of *T. gondii* and the ATPase alpha 1 transporter was established by probability calculations. The probability was calculated by dividing the score of the molecule under study by the lowest energy score (p = composite score/minor score) (Supplementary Tables S1–S5), for each algorithm, and at the end an overall average was calculated between the algorithms to generate the enzyme average ((p) Enzyme = ((p) Moldock Score + (p) Plants Score + (p) Vina Score)/3) [33,34]. The sum of the enzyme mean and division by the number of information originated the total probability (Total P).

### 2.6. Alignment of Protein Sequences

The sequences of the two proteins that do not contain 3D structures in the Protein Data Bank [35] were obtained from the GenBank database [36]. These proteins were: Na^+^/K^+^-transporting ATPase alpha 1—*M. musculus* (NP_659149.1) and Na^+^/K^+^-transporting ATPase alpha 1—*H. sapiens* (NP_000695.2). A global alignment was then performed with the sequence of a protein with a known three-dimensional structure, using the Clustal Omega web tool (WMBL-EBI, 2022 https://www.ebi.ac.uk/Tools/msa/clustalo/ accessed on 14 March 2022) [37], which aligns all protein sequences entered by a user. Alignment facilitated the investigation of the active site and the determination of similarity and shared identity between proteins.

### 2.7. Modeling by Homology

Target sequences were obtained as amino acid sequences in FASTA format and were imported from the SWISS-MODEL website (https://swissmodel.expasy.org/ accessed on 14 March 2022) [38]. For each identified mold, the quality was predicted from alignment features such as ProMod3, QMEAN and GMQE. The stereochemical quality of the models was evaluated by the PSVS (protein structure validation software suite) web server (http://psvs-1_5-dev.nesg.org/ accessed on 14 March 2022), using PROCHECK [39]. PROCHECK generates a Ramachandran chart [34,35], which determines the allowed and disallowed regions of the amino acid backbone.

### 2.8. Molecular Dynamics Simulations

Molecular dynamics simulations were performed to estimate the flexibility of interactions between proteins and ligands, using GROMACS 5.0 software (European Union Horizon 2020 Program, Uppsala, Sweden) [40,41]. The protein and ligand topologies were also prepared using the GROMOS96 54a7 force field. The Molecular Dynamics simulation was performed using the SPC water model of point load, extended in a cubic box [42]. The system was neutralized by the addition of ions (Cl^−^ and Na^+^) and minimized, to remove bad contacts between complex molecules and the solvent. The system was also balanced at 300 K, using the 100 ps V-rescale algorithm, represented by NVT (constant pressure particles and temperature), up to 100 ps. DM simulations were performed in 5,000,000 steps, at 10 ns. To determine the flexibility of the structure and whether the complex is stable close to the experimental structure, RMSD values of all Cα atoms were calculated relative to the starting structures. RMSF values were also analyzed to understand the roles played by residues near the receptor binding site. The RMSD and RMSF graphs were generated using Grace software (Grace Development Team, http://plasma-gate.weizmann.ac.il/Grace/ accessed on 23 June 2022) [43].

## 3. Results

### 3.1. In Vitro Anti-T. gondii Activity and Cytotoxicity against HFF

**Figure 2:**
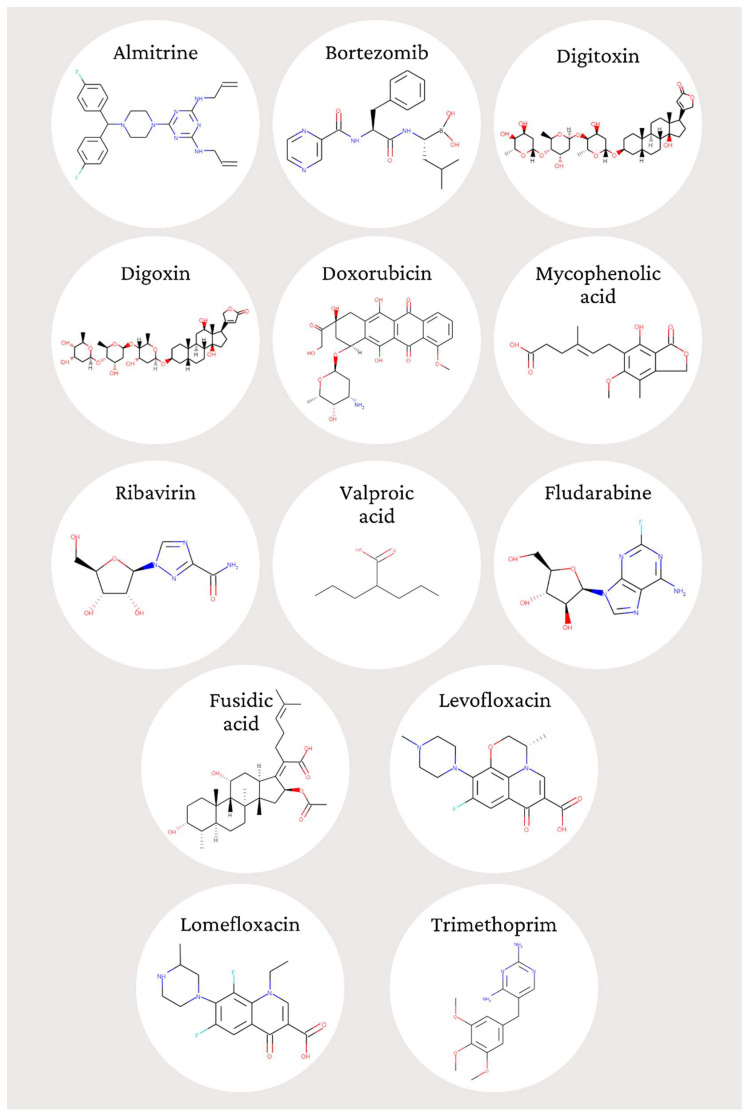
Structures of the 13 compounds tested against *T. gondii* in vitro. The structures were obtained from http://www.ebi.ac.uk/chembl.

We tested 13 compounds that have been in silico selected against *T. gondii* from the MMV foundation’s Pandemic Response Box and COVID Box. Table 1 and Figure 2 show the structures and general characteristics of the tested compounds.

We used a 96-well plate assay based on β-galactosidase expression to estimate the *T. gondii* tachyzoites’ viability. From the 13 tested compounds, three demonstrated anti-*T. gondii* activity at nanomolar range, named almitrine (MMV1804175), bortezomib (MMV009415), and fludarabine (MMV637413), with activity comparable to the reference drug pyrimethamine. A total of ten compounds demonstrated EC_50_ at the micromolar range (digitoxin, digoxin, doxorubicin, fusidic acid, levofloxacin, lomefloxacin, mycophenolic acid, ribavirin, trimethoprim, and valproic acid). The cytotoxicity against mammalian cells was evaluated for the three most active compounds (almitrine, bortezomib, and fludarabine). Almitrine presented the highest selectivity (SI > 47), with a CC_50_ value greater than 20 µM (the higher tested concentration) against HFF. Results concerning the anti-*T. gondii* activity and mammalian cytotoxicity are shown in Table 2.

Based on these results, almitrine was considered a promising anti-*T. gondii* drug candidate. The 13 compounds were subjected to Molecular Docking screening in four proteins for *T. gondii,* and the compound almitrine was subjected to docking simulations with the Na^+^/K^+^-ATPase alpha 1 transporter of *H. sapiens* and *M. musculus*.

### 3.2. In Silico Results

The in silico screening was carried out in two stages, the first corresponding to the evaluation of the probabilities of the compounds against the specific targets for *T. gondii* and the second referring to the screening of the compounds in the ATPase alpha 1 transporter to the species *H. sapiens* and *M. musculus*. Prior to carrying out the Molecular Docking simulations, redocking was performed, aiming to validate the enzymes used in the study. The redocking results (Supplementary Table S1) showed that all targets obtained from the PDB for the organism *T. gondii* had RMSDs below 2.0 Ǻ, indicating that the generated poses of the co-crystallized ligand are correctly positioned at the ligand’s active site.

Docking results were generated using three scoring functions (moldock score, plants score and autodock vina). In addition, the probability of activity in each of the enzymes was calculated. The obtained probability in each algorithm is shown for *T. gondii* enzymes (Supplementary Tables S2–S5) and for the ATPase alpha 1 transporter (Supplementary Tables S6 and S7). The total probability of the compound in the organism was also calculated for *T. gondii* and for the transporter ATPase alpha 1 (Supplementary Tables S8 and S9, respectively). The protein in which the compound obtained probability higher than, or close to, the values obtained by the ligand in at least one scoring function was considered active. Therefore, the ligands selected in the study are co-crystallized in the structure obtained in the PDB library and present experimental validation for the respective enzymes.

For *T. gondii* enzymes, the compound doxorubicin achieved the highest total probability, corresponding to 0.8816 (Supplementary Table S8). Furthermore, the compounds almitrine (0.8461) and bortezomib (0.8383) presented probabilities greater than 0.80, which are close to those obtained for the PDB ligands.

Almitrine presented a significant probability for the ATPase alpha 1 transporter (*H. sapiens*) equivalent to 0.8362 (Supplementary Table S9). Furthermore, it was the most likely compound for the ATPase transporter (*M. musculus*), with *p* = 0.9508, and presented the highest total probability for the two enzymes under study (0.8935). This demonstrates a potency and affinity of this compound for this macromolecule. The molecular coupling of almitrine with transporters for the species *M. musculus* and *H. sapiens* can be seen in Supplementary Tables S4 and S5. The molecular coupling study of almitrine indicated steric, hydrophobic and hydrogen bonding interactions. In addition, it presented residues similar to the positive control tapsigargin, involved the hydrogen interactions of the Arg 551 and Asp 619 residues.

After the analysis of the potential activity of the 13 compounds under study against important *T. gondii* enzymes, Molecular Dynamics simulations were carried out with the compound almitrine to assess the flexibility of the transporting ATPase alpha 1 and the stability of the enzyme interactions in the presence of factors such as solvent, ions, pressure and temperature. This information is important since it complements the docking results and allows one to evaluate whether the compound remains strongly linked to the studied enzymes in the presence of factors that are found in the host organism. To evaluate the stability with the ATPase alpha 1 transporter, the compound almitrine was selected, as it presented the highest total probability for this transporter, taking into account the two species under study: *H. sapiens* and *M. musculus* (Supplementary Table S9). The RMSD was then calculated for the Cα atoms of the complexed enzyme and the structures of each ligand, separately.

**Figure 3:**
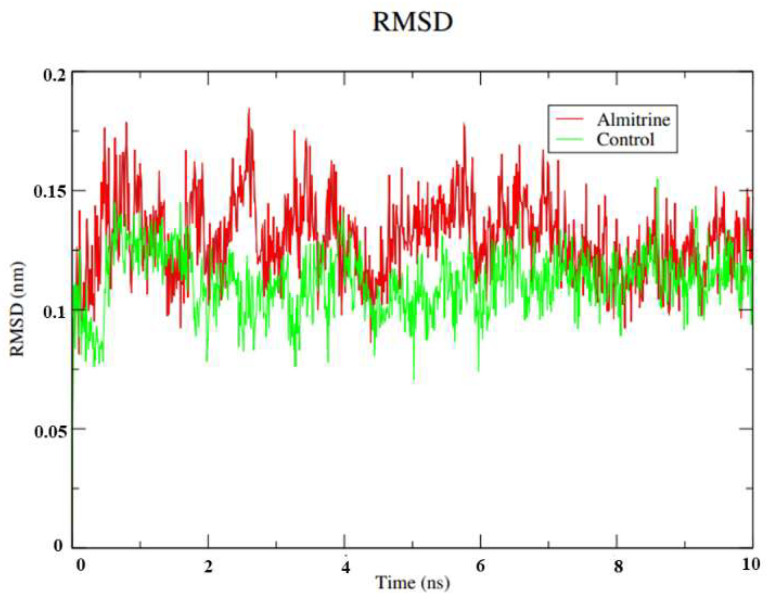
RMSD values of the Cα atoms of almitrine and the control (thapsigargine) with the transporting ATPase aplha 1. Legend: Green: ATPase of *H. sapiens* complexed with thapsigargine; and Red: ATPase of *H. sapiens* complexed with almitrine.

The RMSD analysis of the transporting ATPase alpha 1 of *H. sapiens* with the compound almitrine showed conformations ranging from 0.12 to 0.15 nm in size for 10 ns, with high stability (Figure 3). The stability of this protein is essential to keep compounds bound to the active site. Furthermore, stability prevents the ligand from losing important contacts with the enzyme’s active site.

**Figure 4:**
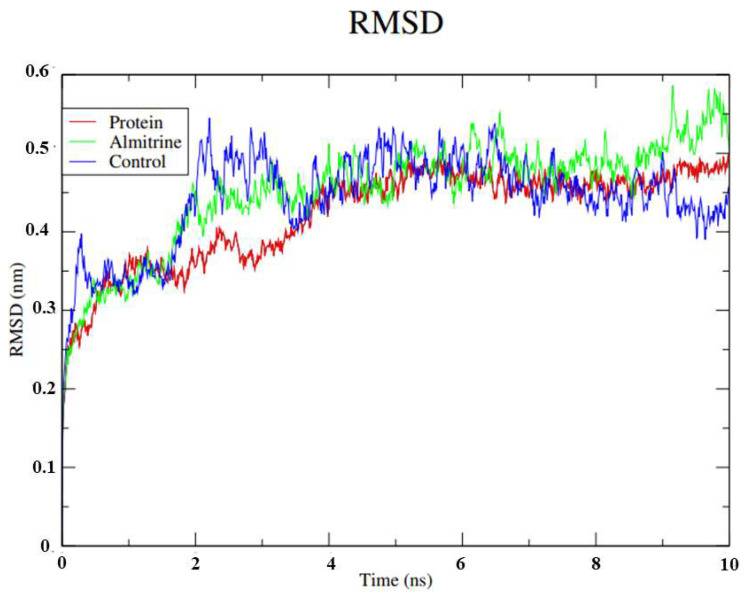
RMSD values for the Cα atoms of the transporting ATPase alpha 1 of *H. sapiens* complexed with almitrine and the control (thapsigargine). Legend: Green: ATPase of *H. sapiens* complexed with almitrine; Blue: ATPase of *H. sapiens* complexed with thapsigargine; and Red: *H. sapiens* transporting ATPase homologous protein.

Regarding the analysis of the flexibility of the ligands through the RMSD calculations of the protein (Figure 4), the profile demonstrated by the isolated protein was similar to the result observed by the control, remaining stable up to 0.4 ns. Almitrine maintained stability up to a certain point, showing a peak in the period from 8.0 to 9.0 ns. Despite the small variation in the protein structure by the peak demonstrated, there was no interference in the structure of the ligands within the active site even if the protein changes its conformation. Therefore, in the presence of solvents, ions and other factors, almitrine was able to establish stronger bonds with the active site.

**Figure 5:**
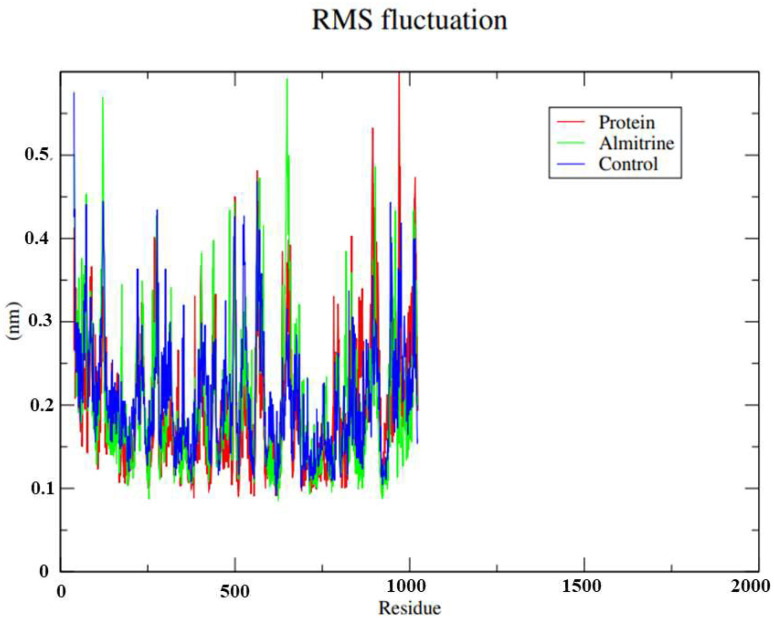
Root-mean-square fluctuation (RMSF) for the Cα atoms of the transporting ATPase of *H. sapiens* alpha 1 complexed with the almitrine and the control thapsigargine. Legend: Green: ATPase of *H. sapiens* complexed with almitrine; Blue: ATPase of *H. sapiens* complexed with the control thapsigargine; and Red: *H. sapiens* transporting ATPase homologous protein.

To understand the flexibility of the residues and amino acids that contribute to the conformational changes in the transporting ATPase alpha 1 of *H. sapiens*, the mean quadratic fluctuation (RMSF) was calculated for each amino acid in each enzyme. High RMSF values suggest greater flexibility. Since amino acids with fluctuations above 0.3 nm contribute to the flexibility of the protein structure, we found that residues at positions 39, 41, 86, 122, 123, 124, 125, 275, 276, 277, 278, 497, 498, 499, 500, 564, 566, 567, 568, 570, 575, 649, 835, 1011, 1012, 1013 and 1016 contribute to conformational changes in the transporting ATPase alpha 1 of *H. sapiens* (Figure 5). We also found that none of the amino acids that affect the structural conformations identified in the transporting ATPase alpha 1 of *H. sapiens* are a component of the active site. This helps almitrine to remain in the active site.

## 4. Discussion

Sarteriale et al., 2014 [13] performed an in silico study based on the proteome of *T. gondii* to identify potential drug candidates for toxoplasmosis therapy. Among the inhibitors previously identified, we selected 13 compounds from the MMV collections to be tested against the parasite in a cell-based assay. We found that the selected compounds were in vitro active against the parasite, with EC_50_ values ranging from 0.22 to 99.69 µM.

The obtained results indicated that this method is valuable and can be used to build enriched compound libraries for in vitro drug testing, which could enable a more efficient use of laboratory resources, as suggested by Sarteriale et al. 2014 [13], bringing the advantage of reduced speed and cost and extra broadness. We also confirmed that the compound collections from MMV are promising sources of anti-*T. gondii* agents.

In our study, diverse antitoxoplasmic compounds were identified, representing the first time that this combined set of compounds has been evaluated against *T. gondii* in vitro.

A total of three compounds showed EC_50_ values against *T. gondii* at the nanomolar range. Two of them (MMV1804175 and MMV009415) belong to the COVID Box and one of them is part of the Pandemic Response Box (MMV637413).

Compound MMV1804175, commercially named almitrine, was the most selective, with an EC_50_ value of 0.424 µM against the parasite and a CC_50_ value higher than 20 µM, the top concentration evaluated. The ratio between the CC_50_ against HFF and the EC_50_ against the parasite resulted in a selectivity index greater than 47. Almitrine is a selective pulmonary vasoconstrictor, which has been proposed as an interesting therapeutic option to manage severe hypoxemia in patients with the Coronavirus 2019 disease [44]. This is the first report about the anti-*T. gondii* activity of almitrine. Previously published work has demonstrated the in vitro activity of this drug against chloroquine-susceptible and chloroquine-resistant *P. falciparum*, with EC_50_ values ranging from 2.6 to 19.8 µM [45]. When almitrine bismesylate was administered to young subjects in single or multiple oral doses, the physiological and blood parameters indicated that the drug was safe at all doses tested, up to 400 mg per day, with symptoms of mild nausea and headache [46].

Bortezomib (MMV009415) is a proteasome inhibitor and antineoplastic agent that is used in the treatment of refractory multiple myeloma and certain lymphomas [47]. The compound was equally effective against drug-sensitive and -resistant *P. falciparum*, blocking its intraerythrocytic development prior to DNA synthesis [48]. Here, we report for the first time the anti-*T. gondii* activity of bortezomib. This compound was the most active, with an EC_50_ value of 0.223 µM against *T. gondii*. However, this compound presented low selectivity, with a CC_50_ value of 0.079 against the mammalian lineage HFF, indicating the need to design possible changes in the chemical structure, aimed at finding more selective analogues.

The purine analogue fludarabine (MMV637413) is an antineoplastic agent used in the therapy of chronic lymphocytic leukemia and in immunosuppressive regimens in preparation of hematopoietic cell transplantation. This small molecule is an analog of the antiviral agent vidarabine and acts interrupting DNA synthesis and inhibiting tumor cell growth. Fludarabine is associated with a low rate of transient serum enzyme elevations during therapy and has only rarely been implicated in cases of clinically apparent acute liver injury [49]. To the best of our knowledge, this is the first report about the anti-parasitic activity of this compound.

Among the ten compounds presenting anti-*T. gondii* activity in the micromolar range, we can highlight doxorubicin, an antibiotic isolated from *Streptomyces peucetius* var. *caesius*. The compound triggers oxidative stress causing cardiotoxicity, which compromises its clinical use as an antineoplastic agent [50]. This anti-*T. gondii* candidate also showed activity against another three parasitic protozoan species, named *C. parvum*, *Trichomonas vaginalis* and *P. falciparum* [51]. To the best of our knowledge, this is the first report about the anti *T. gondii* activity of this compound.

Antibiotics have a history of repurposing success for Apicomplexan parasites and are the conventional treatment for human toxoplasmosis, in the form of pyrimethamine + sulphadiazine, trimethoprim + sulphamethoxazole and pyrimethamine + clindamycin [52]. Other antibiotics with anti-*T. gondii* activity identified in the present work were lomefloxacin, mycophenolic acid, fusidic acid, levofloxacin, and trimethoprim. Mycophenolic acid is an antineoplastic antibiotic derived from various *Penicillium* fungal species. It was previously reported that this drug triggers *T. gondii* extracellular tachyzoites differentiation into cyst-like structures [53]. Fusidic acid, an antibiotic that inhibits the growth of bacteria by preventing the release of translation elongation factor G from the ribosome, has been shown to be effective in tissue culture against *P. falciparum* and *T. gondii* [54]. Trimethoprim is an antimicrobial used to treat and prevent toxoplasmosis and many bacterial infections [55]. Therefore, the in vitro activity of this drug against *T. gondii* is not a novelty. Lomefloxacin is used to treat bacterial infections including bronchitis and urinary tract infections [56]. Levofloxacin is an antibacterial drug with a broad spectrum of activity. This drug diffuses through the bacterial cell wall and acts by inhibiting DNA gyrase (bacterial topoisomerase II), leading to blockage of bacterial cell growth [57]. The in vitro anti-*T. gondii* activity of lomefloxacin and levofloxacin is reported here for the first time.

Digitoxin is a lipid soluble cardiac glycoside that inhibits the plasma membrane Na^+^/K^+^-ATPase, with anticancer effects when used at therapeutic concentrations [58]. In addition, digoxin is a cardiac glycoside long used to treat congestive heart failure and has been found more recently to show anticancer activity [59]. Ribavirin is an inhibitor of the hepatitis C virus polymerase with a broad spectrum of activity against DNA and RNA viruses [60]. To the best of our knowledge, the in vitro anti-*T. gondii* activity of digitoxin, digoxin and ribavirin is first reported here. Valproic acid, a mood-stabilizing and antipsychotic drug, presents efficacy against chronic *T. gondii* infection, as previously demonstrated [61].

Among the three compounds presenting anti-*T. gondii* activity at nanomolar range, we consider almitrine to be the most promising, since this compound showed in vitro selective anti-*T. gondii* activity and presents good oral availability and low human toxicity. The future evaluation of the efficacy of almitrine in *T. gondii*-infected animals is encouraging.

## 5. Conclusions

Promising anti-*T. gondii* candidates were identified and previously published in silico data was confirmed, indicating that this is a useful tool in the search for active compounds in the target-based drug development process. In addition, we suggest that almitrine represents a lead compound against *T. gondii*, which may be useful for antitoxoplasmic chemotherapy.

The 13 selected compounds showed interaction with specific enzymes of *T. gondii*, whilst the compounds almitrine, bortezomib, digoxin, digitoxin, doxorubicin, mycophenolic acid, ribavirin, fludarabine and fusidic acid presented greater affinity than the ligands under study for the selected mechanisms. Almitrine showed a lower score than the positive control tapsigargin, regarding the Na+/K+ ATPase transporter of *H. sapiens* and *M. musculus* referring to the Plantscore algorithm. In addition, almitrine showed interactions such as the positive control tapsigargin, thus indicating a possible mechanism of action of this compound.

## Figures and Tables

**Table 1:** General characteristics of the 13 compounds tested against *T gondii* in vitro.

MMV Code ^a^	Compound (Trivial Name)	Molecular Formula ^b^	Mol wt ^b^	aLogP ^b^	Rule of Five ^b^
MMV1804175	Almitrine	C_26_H_29_F_2_N_7_	477.5	6.09	3
MMV009415	Bortezomib	C_19_H_25_BN_4_O_4_	384.2	2.14	4
MMV002436	Digitoxin	C_41_H_64_O_13_	764.9	3.11	2
MMV002832	Digoxin	C_41_H_64_O_14_	780.9	2	1
MMV004066	Doxorubicin	C_27_H_29_NO_11_	543.5	−0.05	1
MMV003219	Mycophenolic acid	C_17_H_20_O_6_	320.3	3.16	4
MMV001439	Ribavirin	C_8_H_12_N_4_O_5_	244.2	−2.75	4
MMV003305	Valproic acid	C_8_H_16_O_2_	144.2	2.75	4
MMV637413	Fludarabine	C_10_H_12_FN_5_O_4_	285.2	−1.32	4
MMV1578575	Fusidic acid	C_31_H_48_O_6_	516.7	5.1	2
MMV687798	Levofloxacin	C_18_H_20_FN_3_O_4_	361.4	−1.38	4
MMV002350	Lomefloxacin	C_17_H_19_F_2_N_3_O_3_	387.8	−0.83	4
MMV000028	Trimethoprim	C_14_H_18_N_4_O_3_	290.3	1.55	4

^a^ Compounds are named by their MMV identifier codes. ^b^ Molecular formula, molecular weight (Mol wt), aLogP values, and information about rule of five were obtained from the Pandemic Response Box and COVID Box supporting information.

**Table 2:** In vitro activity of the selected compounds against *T. gondii*, with pyrimethamine as the reference drug.

Compound	EC_50_ (µM) ^a^	CC_50_ (µM) ^b^	SI ^c^
Almitrine	0.424	>20	>47
Bortezomib	0.223	0.079	0.35
Digitoxin	5.66	n.d.	n.d.
Digoxin	42.59	n.d.	n.d.
Doxorubicin	2.39	n.d.	n.d.
Mycophenolic acid	8.06	n.d.	n.d.
Ribavirin	83.31	n.d.	n.d.
Valproic acid	99.61	n.d.	n.d.
Fludarabine	0.75	2.140	2.85
Fusidic acid	16.70	n.d.	n.d.
Levofloxacin	70.58	n.d.	n.d.
Lomefloxacin	7.32	n.d.	n.d.
Trimethoprim	7.36	n.d.	n.d.
Pyrimethamine	0.121	n.d.	n.d.

^a^ Half Effective Concentration (EC_50_) against *T. gondii* tachyzoites. ^b^ Half Cytotoxic Concentration (CC_50_) against HFF cells. ^c^ Selectivity indexes (SI) were calculated based on the CC_50_ HFF cells/EC_50_ *T. gondii* ratio. n.d.: not determined.

## Supplementary Materials

The following supporting information can be downloaded at: https://www.mdpi.com/article/10.3390/pharmaceutics14081634/s1. Refs. [62,63] are mentioned in Supplementary Materials.
