# Supplementary material for: Drug Repurposing Based on Protozoan Proteome: In Vitro Evaluation of In Silico Screened Compounds against Toxoplasma gondii
Source: Pharmaceutics. 2022 Aug 5;14(8):1634. doi: 10.3390/pharmaceutics14081634 (PMC9414507; doi:10.3390/pharmaceutics14081634)
Supplement: Supplementary file 1 [file pharmaceutics-14-01634-s001.zip › pharmaceutics-1754912-supplementary.pdf]

pharmaceutics

Supplementary Material

Drug repurposing based on protozoan proteome: *In vitro* evaluation of *in silico* screened compounds against *Toxoplasma gondii*

Débora Chaves Cajazeiro[1], Paula Pereira Marques Toledo[1], Natália Ferreira de Sousa[2], Marcus Tullius Scotti[2], Juliana Quero Reimão[1]

[1] Departamento de Morfologia e Patologia Básica, Faculdade de Medicina de Jundiaí, Jundiaí, Brazil; dcajazeiro@gmail.com; marquestoledo.paula@gmail.com; julianareimao@g.fmj.br
[2] Programa de Pós-graduação em Produtos Naturais e Sintéticos Bioativos (PgPNSB), Instituto de Fármacos e Medicamentos (IPeFarm). Universidade Federal da Paraíba, Campus I, Cidade Universitária, João Pessoa PB 58.051-900, Brazil; nferreiradesousa.nfs@gmail.com; mtscotti@ccae.ufpb.br

1. Probability calculations

Supplementary Table S1. RMSD values for the proteins selected in the study.

Protein	PDB binder	RMSD
Thymidylate synthase	2-amino-5-(phenylsulfanyl)-3,9-dihydro-4H-pyrimido[4,5-b]indol-4-one	0.6046
Purine nucleoside phosphorylase	1,4-dideoxy-4-aza-1-(s)-(9-deazahypoxanthine-9-yl)-d-ribitol	0.2400
enoyl acyl reductase carrier protein	Triclosan	0.1724
Calcium dependent protein kinase 1 (CDKP)	5-amino-1-tert-butyl-3-(quinolin-2-yl)-1H-pyrazole-4-carboxamide	0.6046
ATPase (*H. sapiens)*	Thapsigargine	homology
ATPase (*M. musculus*)	Thapsigargine	homology

Supplementary Table S2. Score values and probability of activity of the compounds under study on the enzyme thymidylate synthase.

Compound	Moldock Score	(p) Moldock Score	Plants Score	(p) Plants Score	Vina Score	(p) Vina Score	(p) Enzyme
Almitrine	-121.305	0.8550	-466.218	0.8374	-5.9	0.8939	0.8621
Bortezomib	-128.076	0.9027	-393.94	0.7076	-5.8	0.8787	0.8297
Digitoxin	-117.229	0.8262	-398.544	0.7159	-4.1	0.6212	0.7211
Digoxin	**-141.875**	1	-357.979	0.6430	**-6.6**	1	0.8810
Doxorubicin	-137.097	0.9663	-494.219	0.8877	-6.1	0.9242	**0.9261**
Mycophenolic Acid	-100.335	0.7072	-353.844	0.6356	-5.2	0.7878	0.7102
Ribavirin	-88.615	0.6245	-346.587	0.6225	-5.8	0.8787	0.7086
Valproic acid	-50.5341	0.3561	-137.455	0.2469	-4.3	0.6515	0.4182
Fludarabine	-107.242	0.7558	-431.579	0.7752	-6.3	0.9545	0.8285
Fusidic acid	-133.48	0.9408	-416.569	0.7483	-6	0.9090	0.8660
Levofloxacin	-63.4513	0.4472	-422.531	0.7590	-5.7	0.8636	0.6899
Lomefloxacin	-83.5192	0.5886	-394.68	0.7089	-6.5	0.9848	0.7608
Trimethoprim	-67.8519	0.4782	-363.451	0.6528	-5	0.7575	0.6295
PDB Ligand	-87.1878	0.6145	**-556.682**	1	-6.5	0.9848	0.8664

Legend: In bold are the best results.

Supplementary Table S3. Score values and probability of activity of the compounds under study on the enzyme purine nucleoside phosphorylase.

Compound	Moldock Score	(p) Moldock Score	Plants Score	(p) Plants Score	Vina Score	(p) Vina Score	(p) Enzyme
Almitrine	-127.911	0.8382	-432.123	0.7609	-6.5	0.7386	0.7792
Bortezomib	-129.914	0.8513	-393.727	0.6932	**-8.8**	1	0.8482
Digitoxin	-61.7236	0.4044	-255.084	0.4491	-6.8	0.7727	0.5421
Digoxin	9.6641	0	-267.169	0.4704	-6.5	0.7386	0.4030
Doxorubicin	-114.487	0.7502	-411.35	0.7243	-7.6	0.8636	0.7794
Mycophenolic Acid	-112.401	0.7365	-383.293	0.6749	-7.8	0.8863	0.7659
Ribavirin	-130.377	0.8543	-456.559	0.8039	-7.4	0.8409	0.8330
Valproic acid	-69.7007	0.4567	-159.358	0.2806	-5.3	0.6022	0.4465
Fludarabine	36.2639	0	119.305	0	**-8.8**	1	0.3333
Fusidic acid	-115.744	0.7584	-383.261	0.6748	-5.1	0.5795	0.6709
Levofloxacin	-84.637	0.5546	-423.041	0.7449	-8.4	0.9545	0.7513
Lomefloxacin	-87.4131	0.5728	-390.42	0.6874	-8.5	0.9659	0.7420
Trimethoprim	-118.061	0.7736	-433.763	0.7637	-6.8	0.7727	0.7700
PDB Ligand	**-152.601**	1	**-567.905**	1	-7.6	0.8636	**0.9545**

Legend: In bold are the best results.

Supplementary Table S4. Score values and probability of activity of the compounds under study on the enzyme enoyl-acyl carrier protein reductase.

Compound	Moldock Score	(p) Moldock Score	Plants Score	(p) Plants Score	Vina Score	(p) Vina Score	(p) Enzyme
Almitrine	-151.561	0.9670	**-567.128**	1	-9.7	0.8584	**0.9418**
Bortezomib	**-156.727**	1	-469.468	0.8277	-9	0.7964	0.8747
Digitoxin	-131.453	0.8387	-431.832	0.7614	-10.9	0.9646	0.8549
Digoxin	-99.8765	0.6372	-485.386	0.8558	**-11.3**	1	0.8310
Doxorubicin	-129.296	0.8249	-559.333	0.9862	-10.1	0.8938	0.9016
Mycophenolic Acid	-91.8875	0.5862	-419.221	0.7392	-8.6	0.7610	0.6955
Ribavirin	-78.9661	0.5038	-473.045	0.8341	-6.8	0.6017	0.6465
Valproic acid	-47.3616	0.3021	-182.668	0.3220	-5.4	0.4778	0.3673
Fludarabine	-110.798	0.7069	-70.4961	0.1243	-7.9	0.6991	0.5101
Fusidic acid	-108.192	0.6903	-480.38	0.8470	-9	0.7964	0.7779
Levofloxacin	-64.011	0.4084	-561.311	0.9897	-9.9	0.8761	0.7580
Lomefloxacin	-74.5413	0.4756	-529.004	0.9327	-9	0.7964	0.7349
Trimethoprim	-68.9302	0.4398	-505.728	0.8917	-7.6	0.6725	0.6680
PDB Ligand	-95.7978	0.6112	-558.059	0.9840	-8.2	0.7256	0.7736

Legend: In bold are the best results.

Supplementary Table S4. Score values and probability of activity of the compounds under study on the enzyme calcium dependent protein kinase 1.

Compound	Moldock Score	(p) Moldock Score	Plants Score	(p) Plants Score	Vina Score	(p) Vina Score	(p) Enzyme
Almitrine	-98.5143	0.7437	-446.347	0.7790	-8.2	0.8817	0.8014
Bortezomib	-125.787	0.9496	-363.957	0.6352	-7.6	0.8172	0.8006
Digitoxin	-103.47	0.7811	-371.161	0.6478	**-9.3**	1	0.8096
Digoxin	**-132.462**	1	-426.354	0.7441	-9.3	1	0.9147
Doxorubicin	-119.472	0.9019	-490.765	0.8565	-9.3	1	**0.9195**
Mycophenolic Acid	-103.988	0.7850	-338.773	0.5912	-7.6	0.8172	0.7311
Ribavirin	-114.186	0.8620	-393.585	0.6869	-6.3	0.6774	0.7421
Valproic acid	-58.5146	0.4417	-152.754	0.2666	-4.7	0.5053	0.4045
Fludarabine	-93.8672	0.7086	-62.72	0.1094	-7.2	0.7741	0.5307
Fusidic acid	-81.9682	0.6188	-326.436	0.5697	-8.3	0.8924	0.6936
Levofloxacin	-80.4464	0.6073	-492.291	0.8592	-8.8	0.9462	0.8042
Lomefloxacin	-93.0443	0.7024	-473.9	0.8271	-7.9	0.8494	0.7930
Trimethoprim	-86.8336	0.6555	-418.238	0.7299	-7.4	0.7956	0.7270
PDB Ligand	-118.728	0.8963	**-572.935**	1	-7.3	0.7849	0.8937

Legend: In bold are the best results.

Supplementary Table S5. Score values and probability of activity of the compounds under study on the enzyme ATPase alpha 1 *H. sapiens*.

Compound	Moldock Score	(p) Moldock Score	Plants Score	(p) Plants Score	Vina Score	(p) Vina Score	(p) Enzyme
Almitrine	-154.256	0.7706	-356.979	0.8875	-7.4	0.8505	0.8362
Bortezomib	-152.357	0.7611	-359.366	0.8935	-6.7	0.7701	0.8082
Digitoxin	**-200.169**	1	-371.178	0.9228	**-8.7**	1	**0.9742**
Digoxin	-186.023	0.9293	-366.97	0.9124	-8	0.9195	0.9204
Doxorubicin	-155.368	0.7761	-383.296	0.9530	-7.1	0.8160	0.8484
Mycophenolic Acid	-124.786	0.6234	-341.962	0.8502	-6	0.6896	0.7211
Ribavirin	-110.893	0.5539	-354.399	0.8811	-5.8	0.6666	0.7006
Valproic acid	-69.8927	0.3491	-128.88	0.3204	-4.1	0.4712	0.3802
Fludarabine	-126.05	0.6297	**-402.191**	1	-6.1	0.7011	0.7769
Fusidic acid	-194.699	0.9726	-378.944	0.9421	-7.4	0.8505	0.9218
Levofloxacin	-106.831	0.5337	-382.294	0.9505	-6.6	0.7586	0.7476
Lomefloxacin	-115.423	0.5766	-332.13	0.8258	-6.7	0.7701	0.7241
Trimethoprim	-109.954	0.5493	-333.977	0.8303	-5.8	0.6666	0.6821
PDB Ligand	-199.549	0.9969	-290.504	0.7223	-6.3	0.7241	0.8144

Legend: In bold are the best results.

Supplementary Table S6. Score values and probability of activity of the compounds under study on the enzyme ATPase alpha 1 *M. musculus*.

Compound	Moldock Score	(p) Moldock Score	Plants Score	(p) Plants Score	Vina Score	(p) Vina Score	(p) Enzyme
Almitrine	-144.571	0.9319	**-80.513**	1	-5.8	0.9206	**0.9508**
Bortezomib	-123.144	0.7938	-64.5417	0.8016	-5.2	0.8253	0.8069
Digitoxin	-152.143	0.9807	-45.7731	0.5685	2.2	0	0.5164
Digoxin	-138.322	0.8916	-43.4735	0.5399	3.4	0	0.4772
Doxorubicin	-133.979	0.8636	-68.7803	0.8542	**-6.3**	1	0.9059
Mycophenolic Acid	-111.742	0.7203	-51.6456	0.6414	-5.3	0.8412	0.7343
Ribavirin	-113.073	0.7288	-52.2021	0.6483	-6.1	0.9682	0.7818
Valproic acid	-78.1745	0.5039	-52.9495	0.6576	-4.8	0.7619	0.6411
Fludarabine	-123.078	0.7933	-56.5048	0.7018	-6.1	0.9682	0.8211
Fusidic acid	-124.063	0.7997	-57.2673	0.7112	-5.4	0.8571	0.7893
Levofloxacin	-91.3501	0.5888	-48.9165	0.6075	-6.1	0.9682	0.7215
Lomefloxacin	-105.194	0.6781	-61.2292	0.7604	-6.1	0.9682	0.8022
Trimethoprim	-93.9306	0.6055	-39.1169	0.4858	-5.3	0.8412	0.6442
PDB Ligand	**-155.129**	1	-45.219	0.5616	-4.4	0.6984	0.7533

Legend: In bold are the best results.

2. Alignment of target sequences

Protein sequence alignment is a tool that helps to verify the similarity and identity of the same protein from different species, or different proteins from the same species. By using this technique, it is possible to analyze the conserved regions and identify common residues of the active site. Furthermore, it is possible to point out structural differences and similarities that can contribute to drug development. Thus, we investigated the amino acids shared between the sequences of the target proteins and the template proteins.

The Na+/K+-transporting ATPase alpha 1 for *H. sapiens* showed a 98.33% identity with the Na+/K+-ATPase in the Na+-bound stat of *Sus scrofa* (**Supplementary Figure S1**). While the Na^+/K^+-transporting ATPase alpha 1 for *M. musculus* showed a 97.06% identity with Na+/K+-ATPase for *Bos taurus* (**Supplementary Figure S2**). The alignment results showed that the target protein sequences contained a high degree of identity and similarity, which allowed the construction of reliable homology models of these proteins.

```
1    MGKGVGRDKYEPAAVSEQGDKKGKKGKKDRDMDELKKEVSMDDHKLSLDELHRKYGTDLS  60
2    MGKGVGRDKYEPAAVSEHGDKK--KAKKERDMDELKKEVSMDDHKLSLDELHRKYGTDLS  58

1    RGLTSARAAEILARDGPNALTPPPTTPEWIKFCRQLFGGFSMLLWIGAILCFLAYSIQAA  120
2    RGLTPARAAEILARDGPNALTPPPTTPEWVKFCRQLFGGFSMLLWIGAILCFLAYGIQAA  118

1    TEEEPQNDNLYLGVVLSAVVIITGCFSYYQEAKSSKIMESFKNMVPQQALVIRNGEKMSI  180
2    TEEEPQNDNLYLGVVLSAVVIITGCFSYYQEAKSSKIMESFKNMVPQQALVIRNGEKMSI  178

1    NAEEVVVGDLVEVKGGDRIPADLRIISANGCKVDNSSLTGESEPQTRSPDFTNENPLETR  240
2    NAEEVVVGDLVEVKGGDRIPADLRIISANGCKVDNSSLTGESEPQTRSPDFTNENPLETR  238

1    NIAFFSTNCVEGTARGIVVYTGDRTVMGRIATLASGLEGGQTPIAAEIEHFIHIITGVAV  300
2    NIAFFSTNCVEGTARGIVVYTGDRTVMGRIATLASGLEGGQTPIAAEIEHFIHIITGVAV  298

1    FLGVSFFILSLILEYTWLEAVIFLIGIIVANVPEGLLATVTVCLTLTAKRMARKNCLVKN  360
2    FLGVSFFILSLILEYTWLEAVIFLIGIIVANVPEGLLATVTVCLTLTAKRMARKNCLVKN  358

1    LEAVETLGSTSTICSDKTGTLTQNRMTVAHMWFDNQIHEADTTENQSGVSFDKTSATWLA  420
2    LEAVETLGSTSTICSDKTGTLTQNRMTVAHMWSDNQIHEADTTENQSGVSFDKTSATWLA  418

1    LSRIAGLCNRAVFQANQENLPILKRAVAGDASESALLKCIELCCGSVKEMRERYAKIVEI  480
2    LSRIAGLCNRAVFQANQENLPILKRAVAGDASESALLKCIELCCGSVKEMRERYTKIVEI  478

1    PFNSTNKYQLSIHKNPNTSEPQHLLVMKGAPERILDRCSSILLHGKEQPLDEELKDAFQN  540
2    PFNSTNKYQLSIHKNPNTAEPRHLLVMKGAPERILDRCSSILIHGKEQPLDEELKDAFQN  538

1    AYLELGGLGERVLGFCHLFLPDEQFPEGFQFDTDDVNFPIDNLCFVGLISMIDPPRAAVP  600
2    AYLELGGLGERVLGFCHLFLPDEQFPEGFQFDTDDVNFPLDNLCFVGLISMIDPPRAAVP  598

1    DAVGKCRSAGIKVIMVTGDHPITAKAIAKGVGIISEGNETVEDIAARLNIPVSQVNPRDA  660
2    DAVGKCRSAGIKVIMVTGDHPITAKAIAKGVGIISEGNETVEDIAARLNIPVSQVNPRDA  658

1    KACVVHGSDLKDMTSEQLDDILKYHTEIVFARTSPQQKLIIVEGCQRQGAIVAVTGDGVN  720
2    KACVVHGSDLKDMTSEQLDDILKYHTEIVFARTSPQQKLIIVEGCQRQGAIVAVTGDGVN  718

1    DSPALKKADIGVAMGIAGSDVSKQAADMILLDDNFASIVTGVEEGRLIFDNLKKSIAYTL  780
2    DSPASKKADIGVAMGIAGSDVSKQAADMILLDDNFASIVTGVEEGRLIFDNLKKSIAYTL  778

1    TSNIPEITPFLIFIIANIPLPLGTVTILCIDLGTDMVPAISLAYEQAESDIMKRQPRNPK  840
2    TSNIPEITPFLIFIIANIPLPLGTVTILCIDLGTDMVPAISLAYEQAESDIMKRQPRNPK  838

1    TDKLVNERLISMAYGQIGMIQALGGFFTYFVILAENGFLPIHLLGLRVDWDDRWINDVED  900
2    TDKLVNEQLISMAYGQIGMIQALGGFFTYFVILAENGFLPIHLLGLRVNWDDRWINDVED  898

1    SYGQQWTYEQRKIVEFTCHTAFFVSIVVVQWADLVICKTRRNSVFQQGMKNKILIFGLFE  960
2    SYGQQWTYEQRKIVEFTCHTPFFVTIVVVQWADLVICKTRRNSVFQQGMKNKILIFGLFE  958

1    ETALAAFLSYCPGMGVALRMYPLKPTWWFCAFPYSLLIFVYDEVRKLIIRRRPGGWVEKE  1020
2    ETALAAFLSYCPGMGVALRMYPLKPTWWFCAFPYSLLIFVYDEVRKLIIRRRPGGWVEKE  1018

1    TYY  1023
2    TYY  1021
```

Supplementary Figure S1. Sequence alignment of *H. sapiens* Na^+/K^+-transporting ATPase alpha 1 (1) and Na^+/K^+-ATPase template in the Na^+-bound stat of *S. scrofa* (2). Gray regions correspond to non-similar and non-identical amino acids. The red regions correspond only to identical amino acids. The yellow regions are similar amino acids.

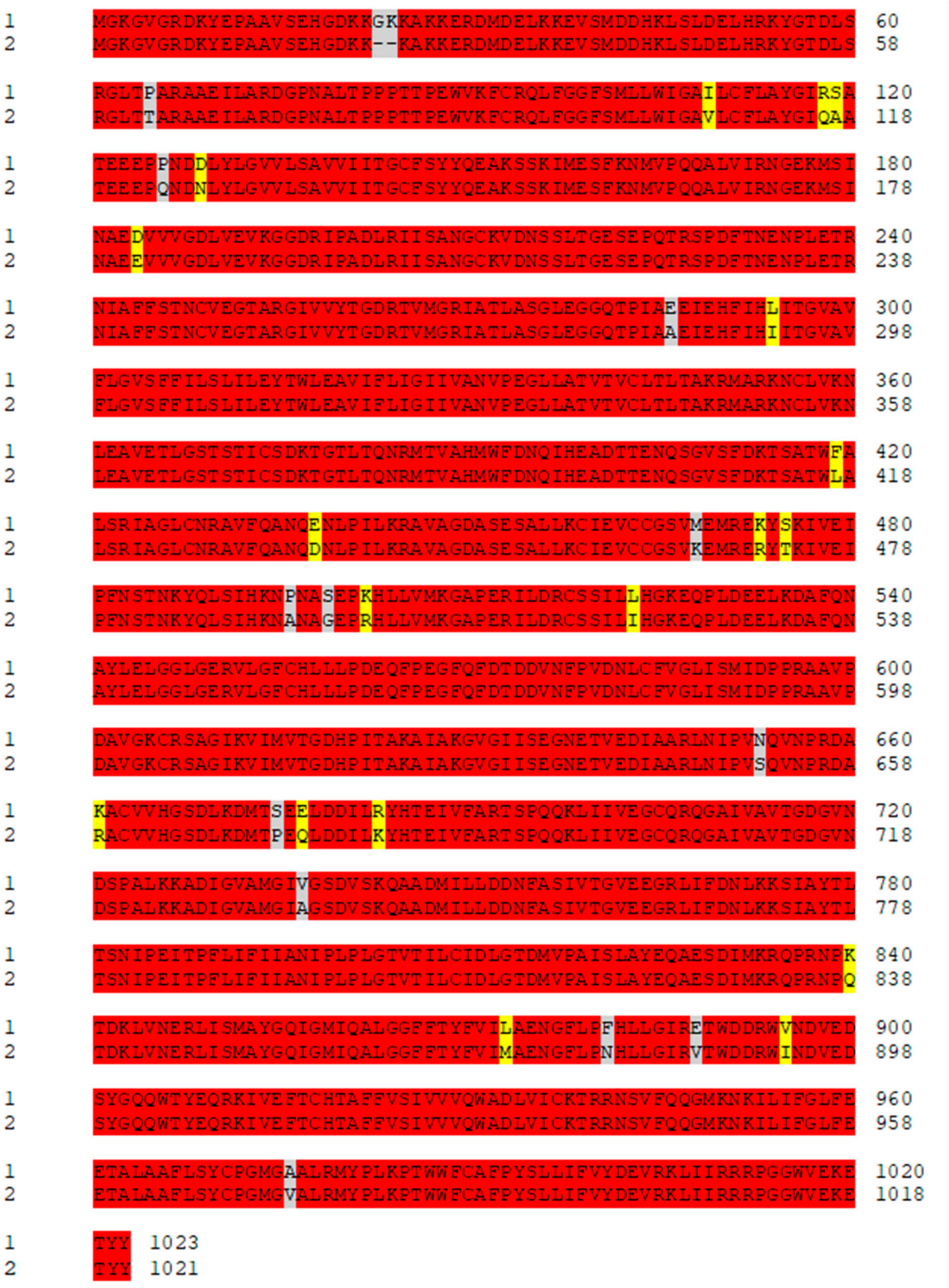

Supplementary Figure S2. Sequence alignment of the *M. musculus* Na^+/K^+-transporting ATPase alpha 1 (1) and the Na^+/K^+-ATPase from bovine template for *B. taurus* (2). Gray regions correspond to non-similar and non-identical amino acids. The red regions correspond only to identical amino acids. The yellow regions are similar amino acids.

3. Modeling by homology

The model for the Na+/K+-ATPase transporter (*H. sapiens*) and the Na+/K+-ATPase transporter (*M. musculus*) was generated by the homology modeling method. The reliability of the models was assessed using the Ramachandran plot, which plots all possible combinations of dihedral angles Ψ (psi) versus φ (phi) for each amino acid in a protein, except glycine, which has no side chains, and the models are considered reliable when more than 90% of the amino acids are present in the allowed and/or favored regions (colored regions of the graph). The blank regions represent outliers, with poor contacts [1]. The Na+/K+-ATPase (*H. sapiens*) transporter model generated showed 91.3% amino acids in the favored regions and 7.5% in the allowed regions (**Supplementary Figure S3A**). While the Na+/K+-ATPase transporter model (*M. musculus*) showed 94.1% of amino acids in the favored regions and 5.7% in the allowed regions (**Supplementary Figure S3B**). Thus, in view of the results, the homology models were considered reliable.

A

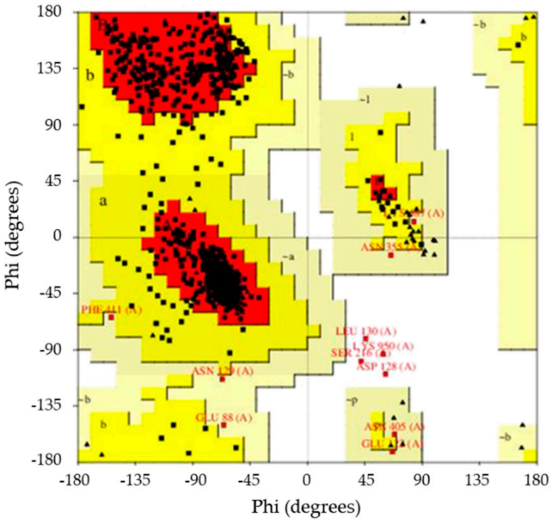

B

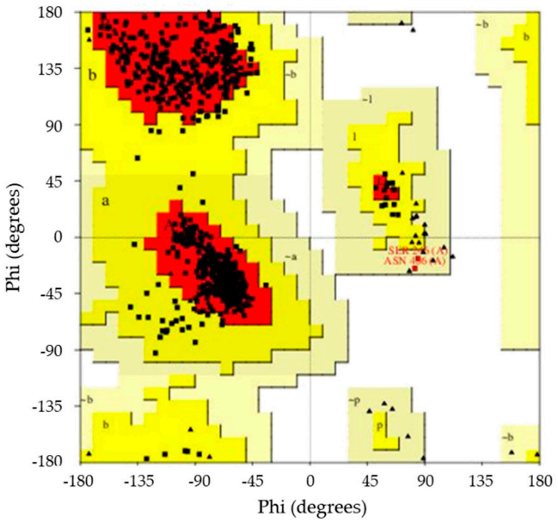

Supplementary Figure S3. Ramachandran plot of the homology model generated for Na+/K+-ATPase transporter (*H. sapiens*) (A) and Na+/K+-ATPase transporter (*M. musculus*) (B). The colored regions represent the allowed and favored regions of secondary structures and the white regions represent the forbidden regions.

4. Molecular docking

The 13 compounds selected for *in vitro* evaluation were submitted to molecular docking screening with specific proteins to evaluate the inhibition of *T. gondii*, which correspond to: thymidyl synthase (PDB: 4KY4), essential for nucleotide synthesis in *T. gondii*; purine nucleoside phosphorylase (PDB: 3MB8), essential for the proliferation of the parasite; enoyl acyl reductase carrier protein (PDB: 2O2S), which aids in the synthesis of the parasite's fatty acid chains; and calcium dependent protein kinase 1 (PDB: 4M48), fundamental in the life cycle of the parasite, as it controls the exocytosis of micronemes, which are specialized organelles that contain various proteins involved in the invasion and egress of the parasite.

The docking results generated by the five scoring functions were validated by redocking the crystallographic ligand with all investigated proteins. The root mean square deviations (RMSDs) of the obtained fit poses were calculated in comparison with the crystal structure. RMSD values of less than 2 Å indicate an optimal degree of screening reliability. Information about the starting structures and redocking validation results is shown in **Supplementary Table S1**.

During the redocking analysis, most of the RMSD values were below 2.0 Å, that is, the generated poses correctly positioned the ligand at the active site. Overall, the programs provided values considered satisfactory for docking validation.

Docking results were generated using three scoring functions, and in addition, the probability of activity in each of the enzymes was calculated. The protein in which the compound obtained a probability higher than, or close to, the values obtained by the ligand in at least one scoring function was considered active. The results of the probability obtained by the four proteins are shown in **Supplementary Table S8.**

Supplementary Table S8. Activity probability values analyzed in the four proteins selected in the study.

Compound	Thymidyl synthase (PDB: 4KY4)	Purine Nucleoside (PDB: 3MB8)	Enoyl Acyl (PDB: 2O2S)	CDKP (PDB: 4M48)	P Total
Almitrine	0.8621	0.7792	**0.9418**	0.8014	0.8461
Bortezomib	0.8297	0.8482	0.8747	0.8006	0.8383
Digitoxin	0.7211	0.5421	0.8549	0.8096	0.7319
Digoxin	0.8810	0.4030	0.8310	0.9147	0.7574
Doxorubicin	**0.9261**	0.7794	0.9016	**0.9195**	**0.8816**
Mycophenolic acid	0.7102	0.7659	0.6955	0.7311	0.7257
Ribavirin	0.7086	0.8330	0.6465	0.7421	0.7326
Valproic acid	0.4182	0.4465	0.3673	0.4045	0.4091
Fludarabine	0.8285	0.3333	0.5101	0.5307	0.5506
Fusidic acid	0.8660	0.6709	0.7779	0.6936	0.7521
Levofloxacin	0.6899	0.7513	0.7580	0.8042	0.7509
Lomefloxacin	0.7608	0.7420	0.7349	0.7930	0.7577
Trimethoprim	0.6295	0.7700	0.6680	0.7270	0.6986
PDB Ligand	0.8664	**0.9545**	0.7736	0.8937	0.8721

Legend: In bold are the best results.

Supplementary Table S8 demonstrates that, for the enzyme thymidylate synthase (PDB: 4KY4), two compounds presented a higher probability than the PDB ligand, namely digoxin (p=0.8810) and doxorubicin (p=0.9261), indicating a higher potency of these compounds for that target. Different to what happened in the first enzyme, for the macromolecule purine nucleoside phosphorylase (PDB: 3MB8), none of the compounds under study presented a higher probability than the ligand, which obtained a probability of 0.9545. The target enoyl acyl reductase carrier protein (PDB: 2O2S) proved to be the macromolecule with the highest affinity with the molecules under study, since it presented the highest number of compounds with higher probability values than the standard PDB ligand under study, totaling six compounds. The highest probability was obtained by almitrine (p=0.9418), followed by doxorubicin (p=0.9016), bortezomib (p=0.8747), digitoxin (p=0.8549), digoxin (p=0.8310) and fusidic acid (p= 0.7779). The PDB ligand obtained a probability of 0.7736. For the enzyme and calcium dependent protein kinase 1 (PDB: 4M48), two molecules under study stood out as more potent, these corresponded to: digoxin (p=0.9147) and doxorubicin (p=0.9195), with the PDB ligand showing probability of 0.8913.

The determination of the individual probabilities of the enzymes made it possible to calculate the total probability (Total P), thus determining the compounds that could possibly be more active for *T. gondii*. The highest Total P obtained with the sum of all individual probabilities was obtained by the compound doxorubicin, corresponding to 0.8816, followed by the sum obtained by the PDB ligands (0.8721). In addition to the compound doxorubicin, the compounds that obtained probabilities greater than 0.80, which corresponded to values close to those obtained for the PDB ligands under study, corresponded to almitrine (0.8461) and bortezomib (0.8383).

The final mechanism comprised the evaluation of the affinity of the compounds under study with the target Na^+/K^+-ATPase. The study of this mechanism is important, since the electrochemical gradient of Na^+ and K^+ ions across the plasma membrane, established by the Na^+/K^+-ATPase or Na^+ pump, is essential for the maintenance of cellular function. This macromolecule belongs to family II of P-type ATPases that transport abundant Ca^{2+} and Na^+ ions in exchange for H^+ or K^+. Structurally, the Na^+/K^+-ATPase pump is composed of a α-catalytic subunit present in all P-type ATPases, in addition to a β-regulatory subunit, which is exclusive to Na/K and H/K ATPase [2]. The activity probabilities obtained by the compounds under study with the Na^+/K^+-ATPase for *H. sapiens* and *M. musculus* are shown in **Supplementary Table S9**.

Supplementary Table S9. Activity probability values analyzed in the ATPase alpha 1 transporter of *H. sapiens* and *M. musculus*.

Compound	ATPase (*H. sapiens*)	ATPase (*M. musculus*)	Total P
Almitrine	0.8362	**0.9508**	**0.8935**
Bortezomib	0.8082	0.8069	0.8076
Digitoxin	**0.9742**	0.5164	0.7453
Digoxin	0.9204	0.4772	0.6988
Doxorubicin	0.8484	0.9059	0.8772
Mycophenolic acid	0.7211	0.7343	0.7277
Ribavirin	0.7006	0.7818	0.7412
Valproic acid	0.3802	0.6411	0.5107
Fludarabine	0.7769	0.8211	0.7990
Fusidic acid	0.9218	0.7893	0.8556
Levofloxacin	0.7476	0.7215	0.7345
Lomefloxacin	0.7241	0.8022	0.7632
Trimethoprim	0.6821	0.6442	0.6631
Thapsigargin	0.8144	0.7533	0.7838

Legend: In bold are the best results.

According to **Supplementary Table S9**, it is possible to see that, for the ATPase alpha 1 *H. sapiens* transporter, five compounds were potentially more potent for this enzyme, these corresponding to: bortezomib (p=0.8082), almitrine (p=0.8362), doxorubicin (p=0.8484), digoxin (p=0.9204) and digitoxin, which corresponds to the highest probability compound (p=0.9742). The positive control tapsigargin had a probability of 0.8144. In a similar way to the transporter from the species *H. sapiens* to the species *M. musculus*, a significant amount of compounds showed affinity for this target, corresponding to seven compounds, with the highest probability obtained by almitrine (0.9508). The other compounds potentially active for this target were, respectively, doxorubicin (p=0.9059), fludarabine (p=0.8211), bortezomib (p=0.8069), lomefloxacin (p=0.8022), fusidic acid (p=0.7893) and ribavirin (p=0.7818). The positive control tapsigargin had a probability of 0.7533.

The compound almitrine presented a significant probability for the ATPase alpha 1 transporter (*H. sapiens*) equivalent to 0.8362. Furthermore, it was the most likely compound for the ATPase transporter (*M. musculus*) with p=0.9508, and presented the highest total probability resulting from the calculated mean for the two enzymes under study with a total of 0.8935. This demonstrates a potency and affinity of this compound for this macromolecule. The molecular coupling of almitrine with transporters for the species *M. musculus* can be seen in **Supplementary Figure S4**.

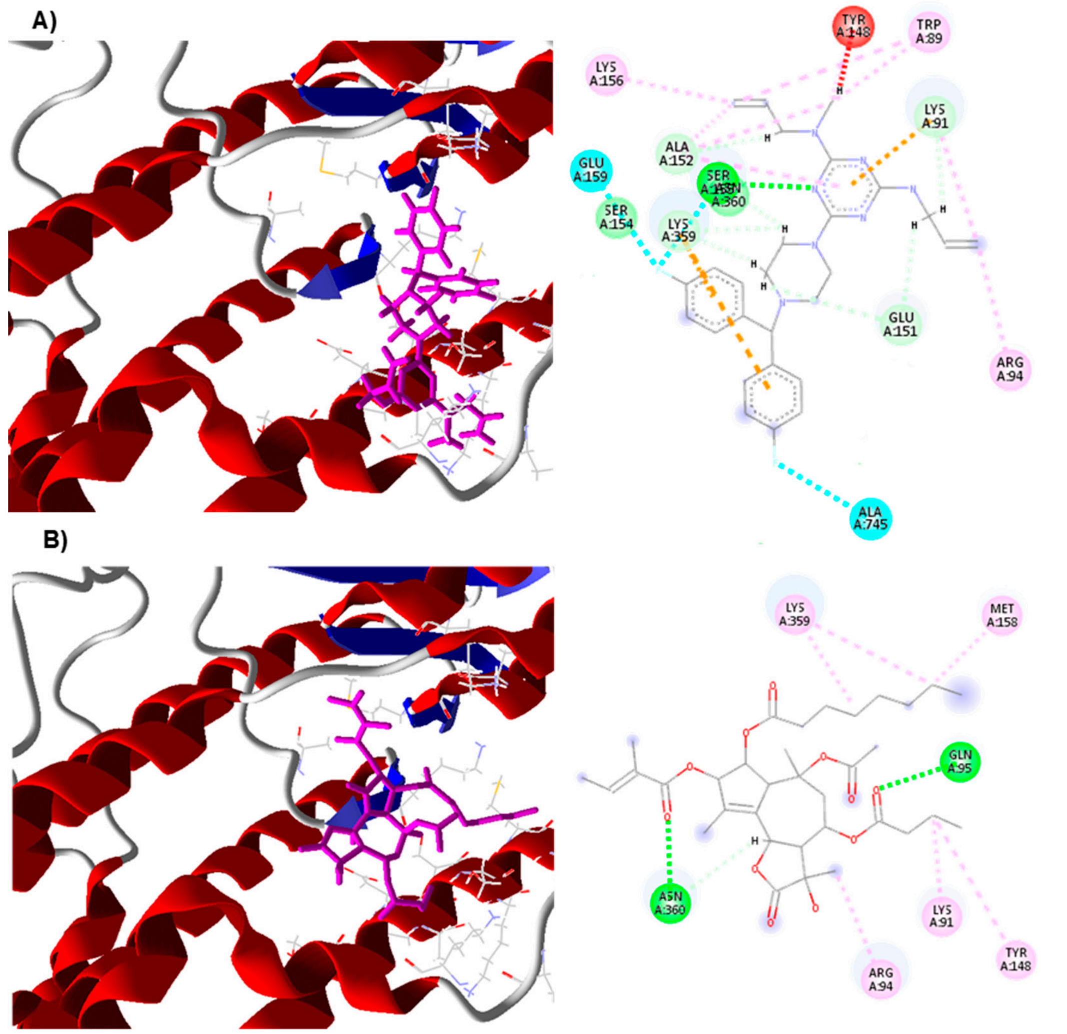

Supplementary Figure S4. Molecular coupling of almitrine (A) and the positive control tapsigargin (B) with the Na^{+}/K^{+}-transporting ATPase alpha 1 (*M. musculus*).
Legend: Green and blue (hydrogen interaction), pink (hydrophobic interaction), red and orange (steric interaction). Residues: Tyr (tyrosine), Trp (tryptophan), Lys (lysine), Glu (glutamic acid), Ala (alanine), Ser (serine), Met (methionine), Gln (glutamine), Asn (asparagine) and Arg (arginine).

The molecular coupling for the transporter referring to *M. musculus* demonstrated the occurrence of the three types of interactions, which correspond to steric interactions (red), hydrophobic interactions (pink) and hydrogen bonds (green and blue). Specifically for almitrine, this was the only one to present the three types of interactions, with the steric interaction being the one with the lowest occurrence, this being performed by residues Tyr 146 (one interaction), Lys 91 (one interaction) and Lys 359 (one interaction) . The hydrophobic interactions occurred between the carbon atoms (C) of the compound structure and were established through the amino acids Lys 156 (one interaction), Ala 152 (thee interactions), Trp 89 (two interactions), Lys 91 (one interaction) and Arg 94 (one interaction). The interactions of the hydrogen type were the most prevalent and were established through the atoms of fluorine (F), nitrogen (N) and hydrogen (H) of the structure of the compound under study, these being established through the residues Glu 151 (two interactions), Ala 745 (one interaction), Lys 359 (one interaction), Ser 155 (two interactions), Glu 159 (one interaction), Ala 152 (one interaction) and Lys 91 (one interaction).

Unlike almitrine, the positive control tapsigargine showed only hydrophobic interactions (pink) and hydrogen bonds (green). The hydrogen bonds occurred with the oxygen atoms (O) of the carbonyl group of the compound under study. The residues responsible for the interactions corresponded to Gln 95 (one interaction) and Asn 360 (two interactions). Hydrophobic interactions were the most prevalent and occurred through amino acids Met 158 (one interaction), Lys 359 (two interactions), Tyr 148 (one interaction), Lys 91 (one interaction) and Arg 94 (one interaction). The similarity between the residue Lys 91 occurred through a hydrophobic interaction established by the two compounds. **Supplementary Figure S5** demonstrates the molecular coupling of almitrine and the positive control tapsigargin with the transporter under study for the organism *H. sapiens*.

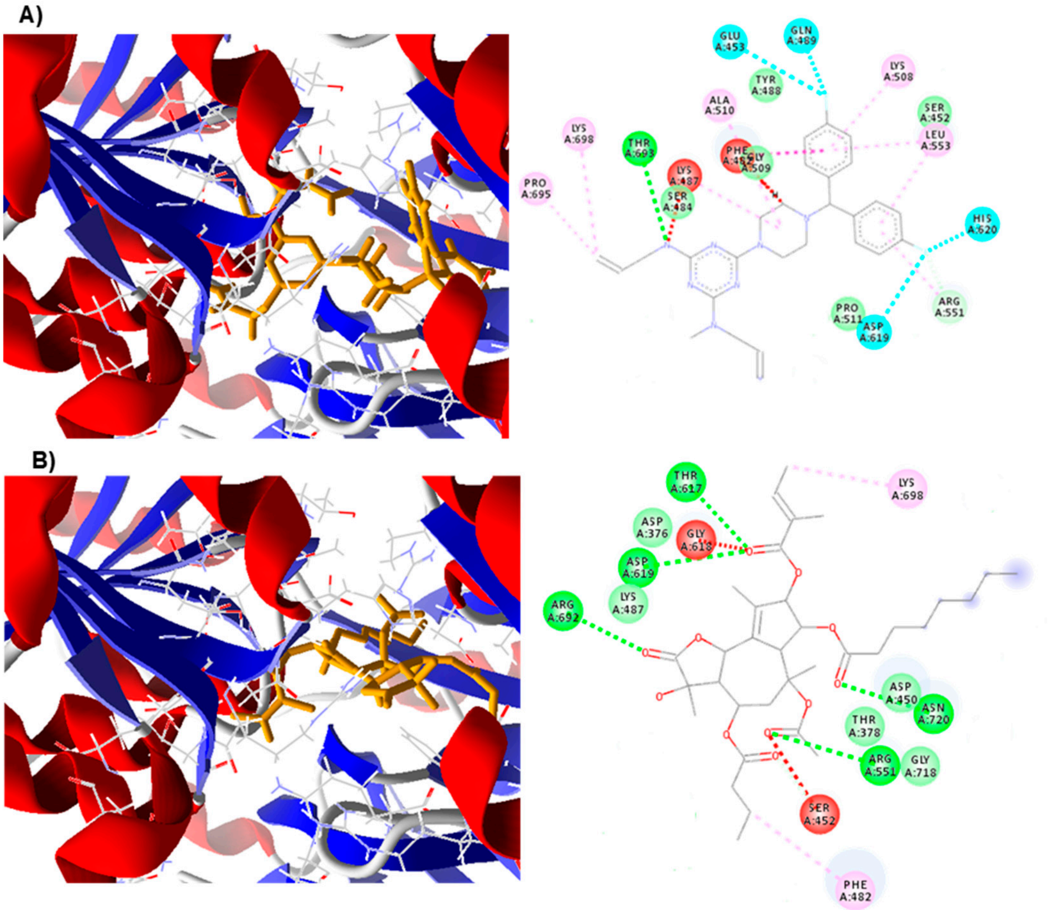

Supplementary Figure S5. Molecular coupling of almitrine (A) and the positive control tapsigargin (B) with the Na^+/K^+-transporting ATPase alpha 1 (*H. sapiens*).
Legend: Green and blue (hydrogen interaction), pink (hydrophobic interaction), red (steric interaction). Residues: Glu (glutamic acid), Gln (glutamine), Lys (lysine), Leu (leucine), His (histidine), Arg (arginine), Asp (aspartic acid), Pro (proline), Thr (threonine), Phe (phenylalanine), Ala (alanine), Gly (glycine), Asn (asparagine) and Ser (serine).

Molecular coupling with the Na^+/K^+-transporting ATPase alpha 1 for *H. sapiens* showed that the two compounds under study presented three types of interactions, namely, hydrogen interactions (green and blue), hydrophobic interactions (pink) and steric interactions (red). For almitrine, six interactions of hydrogen were observed, which were established with the atoms of hydrogen (H), fluorine (F) and nitrogen (N) of the structure of the compound under study. The residues involved were Thr 693 (one interaction), Glu 453 (one interaction), Glen 489 (one interaction), His 620 (one interaction), Arg 551 (one interaction) and Asp 619 (one interaction). The hydrophobic interactions

were established with the carbon atoms (C) of the aromatic rings and the branches, which were carried out by residues Lys 508 (one interaction), Leu 553 (two interactions), Arg 551 (one interaction), Pro 695 (one interaction), Lys 698 (one interaction), Lys 487 (one interaction), Phe 462 (two interactions) and Ala 510 (one interaction). The steric interactions were the least prevalent, being established with the nitrogen atom (N) of the amine group (NH) through the amino acid Lys 487 and with the hydrogen atom (H) of the aromatic ring through the residue Phe 462.

In the positive control tapsigargine, it was observed that the oxygen atoms (O) of the carbonyl groups of the existing organic ester functions were fundamental for the establishment of hydrogen bonds, which are formed by the amino acids Thr 617 (one interaction), Asp 619 (one interaction), Arg 692 (one interaction), Arg 551 (one interaction) and Asn 720 (one interaction). The carbonyl groups also established steric-like interactions through the amino acids Ser 452 (one interaction) and Gly 618 (one interaction). The hydrophobic interactions were established with the groups CH_2 and CH_3 through residues Lys 698 (one interaction) and Phe 482 (one interaction). Similar interactions occurred between almitrine and the positive control, which involved the hydrogen interactions of the Arg 551 and Asp 619 residues. In addition, they also coincided with regard to the residues of the hydrophobic interactions Phe 482 and Lys 698.

References

1. Lovell, S. C.; Davis, I. W.; Arendall, W. B.; De Bakker, P. I. W.; Word, J. M.; Prisant, M. G.; Richardson, J. S.; Richardson, D. C. Structure Validation by Cα Geometry: φ,ψ and Cβ Deviation. *Proteins Struct. Funct. Genet.* **2003**, *50* (3), 437–450. Available online: https://doi.org/10.1002/prot.10286.
2. Hilge, M.; Siegal, G.; Vuister, G. W.; Güntert, P.; Gloor, S. M.; Abrahams, J. P. ATP-Induced Conformational Changes of the Nucleotide-Binding Domain of Na, K-ATPase. *Nat. Struct. Mol. Biol.* **2003**, *10* (6), 468–474.
